# Rapid and Conventional Freezing Conditions of Fish for the Prevention of Human Anisakiasis

**DOI:** 10.14252/foodsafetyfscj.D-24-00015

**Published:** 2025-03-21

**Authors:** Yukihiro Kodo, Rie Murata, Kohji Mori, Jun Suzuki, Kenji Sadamasu

**Affiliations:** Department of Microbiology, Tokyo Metropolitan Institute of Public Health, 3-24-1 Hyakunin-cho, Shinjuku-ku, Tokyo 169-0073, Japan

**Keywords:** *Anisakis* larvae, food poisoning, food safety, freezing conditions, rapid-freezing

## Abstract

In recent years, rapid freezers have been used to freeze and preserve seafood, with advances in freezing technology. However, limited studies have examined the effect of rapid freezing on the viability of *Anisakis* larvae in fish muscle. In this study, freezing experiments were conducted on *Anisakis* larvae alone (bare group) and on larvae embedded in mackerel fish (embedded group) using an air-blast freezer (rapid freezing) as the most popular rapid-freezing method, passing through the zone of maximum ice crystal formation within 30 min, and a natural convection freezer (conventional freezing) set at −20 °C. In the bare group experiments, all larvae died after 8 min of rapid freezing and after more than 2 h of conventional freezing. In the rapid-freezing experiments on the embedded group, only a few larvae were alive when the core temperature of the fish reached −20 °C, whereas all larvae died when the core temperature reached −35 °C. With conventional freezing, only a few larvae were alive for 24 h after freezing at −20 °C. In contrast, all larvae died after freezing at −20 °C for 24 h after the fish core temperature reached −20 °C under both rapid and conventional freezing conditions. In the embedded group, the standard deviation of the time taken for the fish core temperature to reach −20 °C was <15 min for rapid freezing and 171 min for conventional freezing. The results showed that the time taken for the core temperature to reach −20 °C varies by several hours in conventional freezing, depending on the fish size. Thus, the most crucial freezing conditions to avoid anisakiasis are either rapidly freezing the fish to a core temperature of −35 °C or keeping the fish core temperature at −20 °C for at least 24 h.

## 1. Introduction

Anisakiasis is one of the most important food poisonings in Japan and is a gastrointestinal disease caused by the consumption of raw seafood containing third-stage larvae of anisakid nematode^1^^)^. In many fish species, such as skipjack tuna, removal of the ventral muscle and retaining the dorsal muscle alone for raw consumption is considered an effective control method for anisakiasis caused by eating raw fish^2^^)^. However, *Anisakis* larvae have been detected in the dorsal muscle of mackerel^3^^)^; thus, removal of the ventral muscle alone may not be sufficient for preventing food poisoning depending on the fish species. Heat processing of fishery products is the most effective way of killing *Anisakis* larvae. However, if these products are to be eaten raw or almost raw, refrigeration is currently considered the most effective method of preventing anisakiasis, applicable to all fish. The Codex Alimentarius Commission, the European Union, and the Food and Drug Administration have established their own standards for freezing conditions to kill *Anisakis* larvae^4^^,^^5^^,^^6^^)^. In Japan, the Ministry of Health, Labour, and Welfare recommends “freezing fish at −20 °C for at least 24 h;” however, effectiveness in preventing anisakiasis may vary depending on the size of the fish and the performance of the freezing equipment.

Recent advances in freezing technology have enabled the development of equipment that can rapidly freeze food. Currently, several types of rapid freezers are known, such as air-blast freezers and brine freezers, which can pass through the zone of maximum ice crystal formation within 30 min. Air-blast freezers, which circulate air temperatures between −35 °C and −45 °C to freeze them from refrigeration temperature to the desired storage temperature, are the most popular and are often used to freeze fish for consumption as sashimi to preserve its quality. There are several reports of experiments using freezing conditions for fish to kill *Anisakis* larvae^7^^,^^8^^,^^9^^)^. In particular, the freezing conditions for *Anisakis* larvae using a freezer with a double compressor, which has a higher freezing capacity than that of a conventional freezer, were described by Podolska et al.^10^^)^. However, all of these experiments examined the larvae in fish fillets or the abdominal cavity, and no comparative study was performed using a rapid freezer on the larvae in the whole fish muscle. In Japan, mackerel is the primary seafood agent responsible for *Anisakis* food poisoning^2^^)^. In this study, we used chub mackerel (*Scomber japonicus*) and *Anisakis* larvae from these fish to evaluate the freezing conditions necessary to kill *Anisakis* larvae and prevent food poisoning reliably. We examined the relationship between freezing time and the viability of *Anisakis* larvae using both rapid and conventional freezers.

## 2. Material and Methods

### 2.1. Freezing Equipment

An air-blast freezer (KFQ-8A-300B, 3D freezer, manufactured in 2018, KOGASUN, Tokyo, Japan), which can pass the zone of maximum ice crystal formation (−5 °C to −1 °C) within 30 min, was used as the rapid freezer. A natural convection freezer (JF-NC 145F, manufactured in 2018, Haier, Qingdao, China), which can be set from −15 °C to −24 °C, was used at −20 °C as the conventional freezer. Data loggers (SK-L200TII, SATO KEIRYOKI, Tokyo, Japan) were placed in each freezer cabinet to monitor the internal temperature.

### *2.2.* Anisakis *larvae*

The *Anisakis* larvae used in the freezing experiments were collected from the viscera of chub mackerel caught on the Pacific coast of Japan, which are reported to be highly parasitized with *Anisakis simplex* sensu stricto^11^^)^. The collected larvae were examined under a microscope to determine the ventricular length, shape of the caudal end, and presence of a mucron; larvae were morphologically identified as Type I, third-stage larvae, as described previously^12^^,^^13^^)^. The larvae, which were presumed to be *A. simplex* sensu stricto based on the morphological characteristics by Quiazon et al.^14^^)^ were used in the freezing experiments.

### 2.3. Freezing Experiments on Larvae Alone (bare Group)

**Fig. 1.**
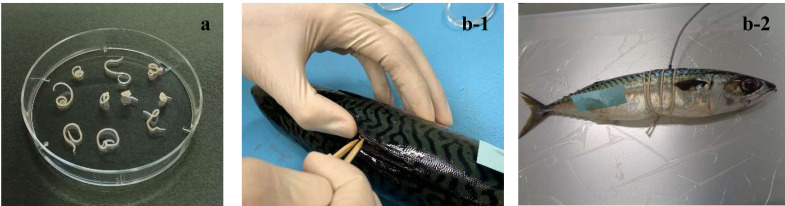
Preparation of (a) bare and (b) embedded groups of *Anisakis* larvae. (a) *Anisakis* larvae were directly placed into disposable Petri dishes. (b-1) A slit was cut near the dorsal fin of the mackerel, and *Anisakis* larvae were embedded in the center of the fish. (b-2) A temperature logger probe was inserted into the slit and secured by wrapping a rope around the fish body.

Ten test larvae were placed in a single sterile disposable Petri dish, and moisture on the larvae was absorbed using a lab wipe (**Fig. 1**). The freezing experiments were conducted twice using 10 larvae each under rapid and conventional freezing conditions. The freezing conditions for the bare group were storage for 2, 5, and 8 min in the rapid-freezing mode of a rapid freezer pre-cooled to −20 °C and freezing for 30 min, 1 h, 2 h, 18 h, and 24 h in a conventional freezer (internal temperature set at −20 °C). Internal temperature was measured by reading displays in the freezers and using data loggers.

### 2.4. Freezing Experiments on Larvae Embedded in Mackerel (embedded Group)

A slit was cut near the dorsal fin of the freshly prepared whole (round) mackerel, and ten larvae and logger probes, measuring core temperature, were embedded in the center of the fish near the spine. A rope was wrapped around the fish body to secure the larvae and probe and prevent the slit from opening (**Fig. 1**). Two-to-five mackerel with embedded larvae (20–50 test larvae) were frozen under rapid and conventional freezing conditions. The rapid-freezing conditions in the rapid-freezing mode of the rapid freezer (pre-cooled to −20 °C) for the embedded group were: reaching the mackerel core temperature of −20 °C (Experiment A), reaching the mackerel core temperature of −35 °C (Experiment B), and storage at −20 °C for 24 h after reaching the mackerel core temperature of −20 °C (Experiment C) (**Table 1**). The freezing conditions for conventional freezing were: storage at −20 °C for 24 h (Experiment D) and storage at −20 °C for 24 h after reaching the mackerel core temperature of −20 °C (Experiment E) (**Table 1**). After the freezing experiment, the mackerel were thawed under running water, and the embedded larvae were collected.

**Table 1.** Freezing conditions for the embedded group for each experiment and size of mackerel used in the freezing experiment

Experiment	Type of freezer	Freezing condition	No. of tested larvae (No. of fish samples)	Fork length (cm) Mean ± SD	Weight (g) Mean ± SD
A	Rapid freezer	Core temperature reaching −20 °C	50 (5)	37.5 ± 2.2	614 ± 100
B	Rapid freezer	Core temperature reaching −35 °C	50 (5)	37.7 ± 2.1	655 ± 68
C	Rapid freezer	Storage at −20 °C for 24 h after reaching core temperature of −20 °C	50 (5)	37.2 ± 2.6	636 ± 96
D	Conventional freezer	Storage at −20 °C for 24 h	20 (2)	34.0 ± 0.0	438 ± 19
E	Conventional freezer	Storage at −20 °C for 24 h after reaching core temperature of −20 °C	50 (5)	35.9 ± 1.6	602 ± 34

SD, standard deviation.

### 2.5. Viability Test for Larvae after Freezing

**Fig. 2.**
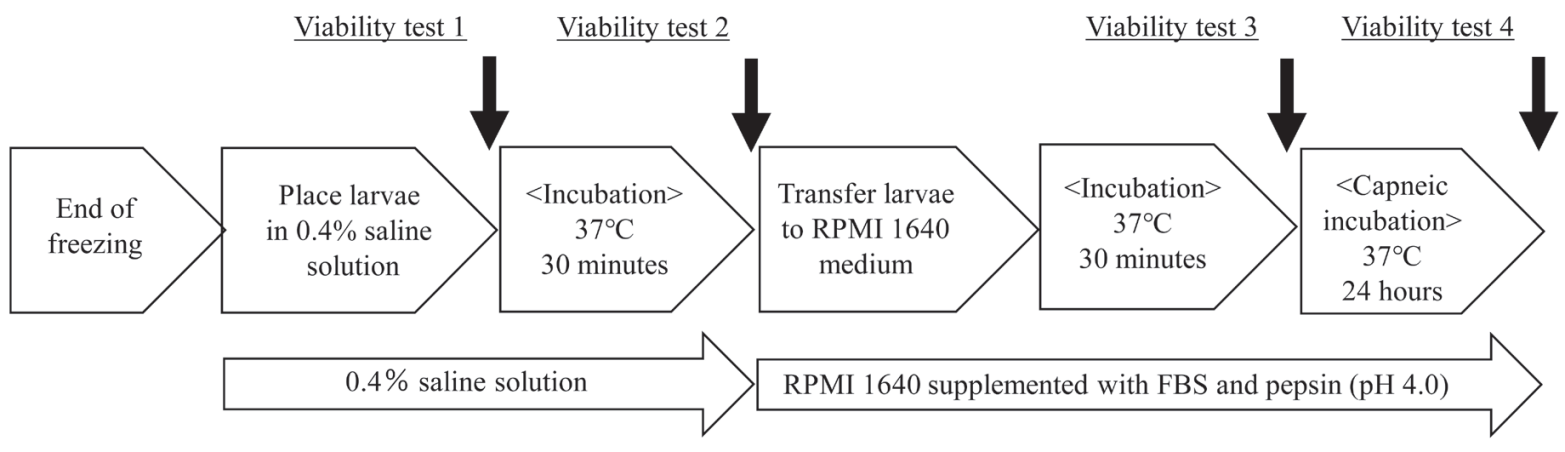
Viability test flowchart. Black arrows indicate the point where the viability test was performed. White arrows indicate the culture medium in which the larvae were incubated.

A viability test for *Anisakis* larvae was conducted using 0.4% saline and 1% pepsin-supplemented RPMI 1640 medium containing 20% heat-inactivated fetal bovine serum (pH 4.0) used to culture *Anisakis* larvae^15^^)^. Viability of the larvae after conducting the aforementioned freezing experiments was determined as follows: after the freezing experiments, the larvae were placed in 0.4% saline, incubated at 37 °C for 30 min, and then transferred to 1% pepsin-supplemented RPMI 1640 medium containing 20% heat-inactivated fetal bovine serum (pH 4.0), and capneic incubation with 5% CO_2_ using AnaeroPack™-CO_2_ (Mitsubishi Gas Chemical, Tokyo, Japan) was performed at 37 °C for 24 h. Larval viability checks were performed at four points: immediately after placing the larvae in 0.4% saline, after incubation in saline at 37 °C for 30 min, after transferring the larvae to RPMI 1640 medium and incubating at 37 °C for 30 min, and after another 24 h of capneic incubation at 37 °C in RPMI 1640 medium. Viability was checked under the stereo microscope, and larvae were considered viable if motility was observed upon stimulation with a bamboo skewer (**Fig. 2**).

## 3. Results

### 3.1. Freezing Experiments on the Bare Group

Under rapid-freezing conditions, more than half of the larvae were alive at a freezing time of 2 min; however, all larvae died at a freezing time of 8 min (**Table 2**). When the freezing time was 5 min, few larvae were alive after 30 min of incubation at 37 °C in 0.4% saline; however, they were dead after 24 h of microaerophilic incubation at 37 °C in RPMI 1640 medium (**Table 2**). Under conventional freezing conditions, few larvae survived when the freezing time was less than 1 h; however, all larvae died when the freezing time was more than 2 h (**Table 2**).

**Table 2.** Viability of *Anisakis* larvae under different freezing conditions

Viability test ^a^	Results of viability test after freezing (No. of motile larvae/Larvae tested)								
	Rapid freezing ^b^			Conventional freezing (−20 °C)					
	2 min	5 min	8 min	5 min	30 min	1 h	2 h	18 h	24 h
Checkpoint 1	10/20	0/20	0/20	17/20	7/20	0/20	0/20	0/20	0/20
Checkpoint 2	11/20	2/20	0/20	17/20	8/20	1/20	0/20	0/20	0/20
Checkpoint 3	14/20	2/20	0/20	20/20	8/20	1/20	0/20	0/20	0/20
Checkpoint 4	13/20	0/20	0/20	17/20	8/20	1/20	0/20	0/20	0/20
Temperature inside the freezer at the end of experiment ^c^	−27.7 °C	−29.6 °C	−32.8 °C						

^a^ Checkpoints 1–4 correspond to "viability test" 1–4 in Fig. 2.

^b^ Time elapsed since the larvae were placed in the freezer pre-cooled at −20 °C and initiation of rapid freezing.

^c^ Average temperature of two experiments.

### 3.2. Freezing Experiments on the Embedded Group

**Fig. 3.**
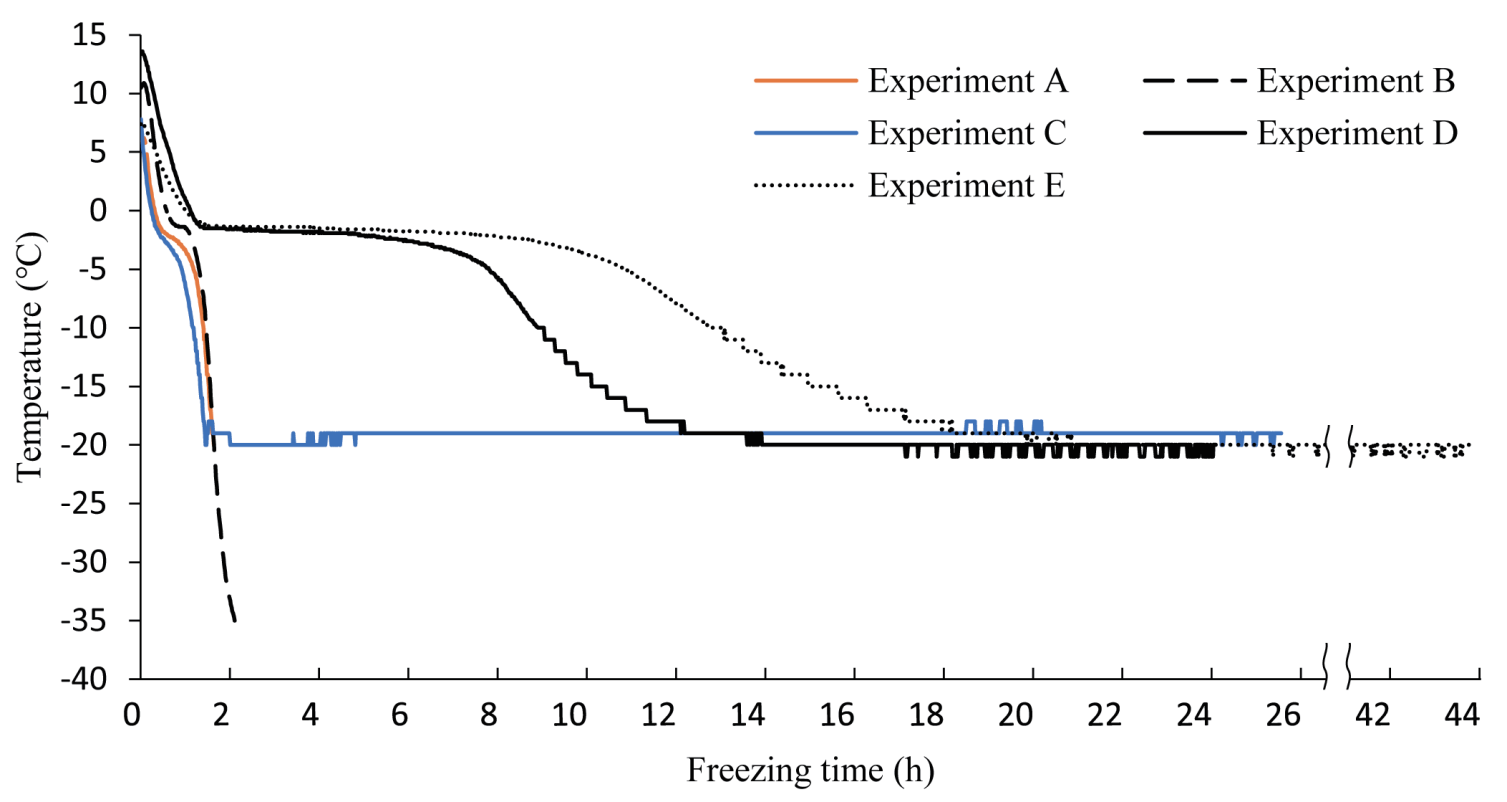
Freezing curves for the embedded group. The curves of samples with the longest freezing times in each experiment were selected. Experiment A: reaching a core temperature of −20 °C (rapid freezing); experiment B: reaching a core temperature of −35 °C (rapid freezing); experiment C: storage at −20 °C for 24 h after the core temperature reaches −20 °C (rapid freezing); experiment D: storage at −20 °C for 24 h (conventional freezer); experiment E: storage at −20 °C for 24 h after the core temperature reaches −20 °C (conventional freezer).

In rapid-freezing experiments, the core temperatures of all mackerel used in the experiments reached −20 °C within 2 h (**Fig. 3**). The standard deviation of the time required for the core temperature to reach the target value was 15 min or less (**Table 3**). One larva was still alive when the core temperature of the mackerel reached −20 °C (**Table 3**; Experiment A); however, all larvae were dead when the core temperature reached −35 °C (**Table 3**; Experiment B). All larvae were dead when stored at −20 °C for 24 h after the core temperature reached −20 °C (**Table 3**; Experiment C).

**Table 3.** Results of freezing experiments on the embedded group and freezing time until the respective core temperature is reached.

Viability test ^a^	Results of viability test after freezing (No. of motile larvae/Larvae tested)				
	Rapid freezing			Conventional freezing	
	Experiment A	Experiment B	Experiment C	Experiment D	Experiment E
Checkpoint 1	0/50	0/50	0/50	0/20	0/50
Checkpoint 2	0/50	0/50	0/50	1/20	0/50
Checkpoint 3	2/50	0/50	0/50	2/20	0/50
Checkpoint 4	1/50	0/50	0/50	2/20	0/50
Time taken to reach core temp. (Mean ± SD)	−20 °C: 81 ± 13 min ^b^	−20 °C: 88 ± 8 min ^b^ −35 °C: 115 ± 9 min ^b^	−20 °C: 80 ± 12 min ^b^	−20 °C: 815 min ^c, d^	−20 °C: 981 ± 171 min ^c^

^a^ Checkpoints 1–4 correspond to "viability test" 1–4 in Fig. 2.

^b^ Time elapsed since the mackerel were placed in the rapid freezer (pre-cooled at −20 °C) and initiation of rapid freezing.

^c^ Time elapsed since the mackerel were placed in a conventional freezer pre-cooled at −20 °C.

^d^ Of the 2 mackerel, freezing time was measured for 1 mackerel.

SD, standard deviation.

In conventional freezing experiments, the mackerel that took the longest to reach a core temperature of −20 °C required approximately 20 h (Fig. 3). The standard deviation of the time required for the core temperature to reach the target temperature was 171 min (Table 3). Only a few larvae were alive when the mackerel was stored at −20 °C for 24 h (Table 3; Experiment D), whereas all larvae were dead when it was stored at −20 °C for an additional 24 h after the core temperature reached −20 °C (Table 3; Experiment E).

## 4. Discussion

The results of the freezing experiments for the bare group showed that all larvae died after 8 min of rapid freezing and after more than 2 h for conventional freezing. When only *Anisakis* larvae are refrigerated, supercooling is released between −10 °C and −30 °C, causing the larvae to freeze and die^16^^)^. In the rapid-freezing experiments, the freezer temperature at the end of the session was below −30 °C for the conditions in which all larvae died. For conventional freezing, it is assumed that the larvae were maintained at −20 °C for an extended period, resulting in their death due to freezing.

In the experiments on the embedded group, only a few larvae survived when the core temperature of the mackerel reached −20 °C through rapid freezing. These results suggest that reaching a core temperature of −20 °C is insufficient to kill larvae in fish, as observed in the bare group experiments. In contrast, all larvae died when the core temperature of the mackerel reached −35 °C. Rapid freezing is considered to cause less damage to cells than conventional freezing, as it results in small ice crystals within cells, presumably inflicting less harm on *Anisakis* larvae. Nevertheless, it was verified that all embedded larvae were killed by lowering the core temperature of the mackerel to −35 °C. The average freezing time needed to reach a core temperature of −35 °C was 115 min, suggesting that rapid freezing may reduce the freezing time required to kill *Anisakis* larvae compared to the time required with conventional freezing. During conventional freezing, two of 20 larvae were alive after the mackerel was stored at −20 °C for 24 h. This result aligns with findings from a previous study on herring^10^^)^ and may be attributed to the time (815 min) required in this experiment for the core temperature to reach −20 °C, which was insufficient freezing time to kill the larvae embedded in the mackerel. In contrast, all larvae were dead when stored at −20 °C for an additional 24 h after reaching a core temperature of −20 °C in both rapid and conventional freezing. These results confirm that simply freezing fish for 24 h at −20 °C is not sufficient to ensure the death of *Anisakis* larvae and that it is important to freeze fish for 24 h after the fish core temperature reaches −20 °C.

The results of these freezing experiments revealed that *Anisakis* larvae in fish muscle can be killed by rapid freezing to achieve a fish core temperature of −35 °C. Rapid freezing is considered effective in preventing anisakiasis, as it reduces the time required to kill *Anisakis* larvae and helps preserve the quality of the seafood. On the contrary, to kill the larvae in fish muscle by freezing at −20 °C, it is necessary to store the fish at this temperature for 24 h after the fish core temperature reached −20 °C in both rapid and conventional freezing. The changes in fish core temperature in the embedded group of conventional freezing showed that the time taken for the fish core to reach the target temperature varied by a few hours. This variation can be attributed to differences in fish size, suggesting the importance of monitoring the core temperature of the fish. However, in addition to fish size, other factors influencing temperature changes in frozen fish include the freezer’s layout, the freezing equipment’s performance, and the frequency with which the doors are opened and closed. Moreover, it is difficult to constantly monitor the core temperature of fish when cooking in restaurants or homes. Therefore, when preparing seafood for raw consumption using conventional freezing, it is important to process the fish into filets before freezing to reduce the core temperature more rapidly and ensure that the fish is frozen for a sufficient duration, considering the time it takes for the core temperature to reach −20 °C.

## References

[1] Arizono N, Yamada M, Tegoshi T, Yoshikawa M. *Anisakis simplex* sensu stricto and *Anisakis pegreffii*: biological characteristics and pathogenetic potential in human anisakiasis. Foodborne Pathog Dis. 2012; 9(6): 517–521. 22545961 10.1089/fpd.2011.1076

[2] Murata R, Suzuki J, Kodo Y, et al. Probable association between *Anisakis* infection in the muscle of skipjack tuna (*Katsuwonus pelamis*) and human anisakiasis in Tokyo, Japan. Int J Food Microbiol. 2021; 337: 108930. 33161348 10.1016/j.ijfoodmicro.2020.108930

[3] Kodo Y, Murata R, Suzuki J, Mori K, Sadamasu K. Prevalence of *Anisakis* larvae in cultured mackerel *Scomber japonicas* in Japan and the relationship between the intensity of *Anisakis* infection in cultured mackerel and fish fatness. Int J Food Microbiol. 2023; 404: 110347. 10.1016/j.ijfoodmicro.2023.110347 37543025

[r4] 4.Food and Agriculture Organization of the United Nations and World Health Organization. Code of practice for fish and fishery products. https://iris.who.int/handle/10665/336524. Published 2020. Accessed August 7, 2024

[r5] 5.Commission Regulation (EU) No 1276/2011 of 8 December 2011 amending Annex III to Regulation (EC) No 853/2004 of the European Parliament and of the Council as regards the treatment to kill viable parasites in fishery products for human consumption. https://eur-lex.europa.eu/eli/reg/2011/1276/oj/eng. Published 2011. Accessed August 7, 2024

[r6] 6.U.S. Department of Health and Human Services Food and Drug Administration. Fish and fishery products hazards and controls guidance. https://www.fda.gov/media/80637/download?attachment. Published 2022. Accessed August 7, 2024

[7] Adams AM, Ton MN, Wekell MM, Mackenzie AP, Dong FM. Survival of *Anisakis simplex* in arrowtooth flounder (*Atheresthes stomia*) during frozen storage. J Food Prot. 2005; 68(7): 1441–1446. 16013383 10.4315/0362-028x-68.7.1441

[8] Lanfranchi AL, Sardella NH. Anisakids survival after microwaving, freezing and salting fish from Argentina. Food Sci Technol Res. 2010; 16(5): 499–504.

[9] Wharton DA, Aalders O. The response of *Anisakis* larvae to freezing. *J Helminthol*. 2002; **76**(4): 363–368. 10.1079/JOH2002149 12498643

[r10] 10.Podolska M, Pawlikowski B, Nadolna-Ałtyn K, Pawlak J, Komar-Szymczak K, Szostakowska B. How effective is freezing at killing *Anisakis simplex, Pseudoterranova krabbei*, and *P. decipiens* larvae? An experimental evaluation of time-temperature conditions. *Parasitol Res*. 2019; **118**(7): 2139–2147. , 10.1007/s00436-019-06339-1 PMC6611746 31098726

[11] Suzuki J, Murata R, Hosaka M, Araki J. Risk factors for human Anisakis infection and association between the geographic origins of Scomber japonicus and anisakid nematodes. Int J Food Microbiol. 2010; 137(1): 88–93. 19892425 10.1016/j.ijfoodmicro.2009.10.001

[12] Shiraki T. Larval nematodes of family Anisakidae (Nematoda) in the northern sea of Japan as a causative agent of eosinophilic phlegmone or granuloma in the human gastrointestinal tract. Acta Med Biol (Niigata). 1974; 22: 57–98.

[13] Murata R, Suzuki J, Sadamasu K, Kai A. Morphological and molecular characterization of *Anisakis* larvae (Nematoda: Anisakidae) in *Beryx splendens* from Japanese waters. *Parasitol Int*. 2011; **60**(2): 193–198. 10.1016/j.parint.2011.02.008 21377540

[14] Quiazon KMA, Yoshinaga T, Ogawa K, Yukami R. Morphological differences between larvae and in vitro-cultured adults of *Anisakis simplex* (sensu stricto) and *Anisakis pegreffii* (Nematoda: Anisakidae). Parasitol Int. 2008; 57(4): 483–489. 18644463 10.1016/j.parint.2008.06.003

[15] Iglesias L, Valero A, Benítez R, Adroher FJ. *In vitro* cultivation of *Anisakis simplex*: pepsin increases survival and moulting from fourth larval to adult stage. *Parasitology.* 2001; **123**(Pt 3): 285–291. 10.1017/s0031182001008423 11578092

[16] Takeuchi M, Matsubara H, Takahashi T, et al. Influence of freezing on the survival of third stage *Anisakis* larvae [In Japanese]. Trans JSRAE. 2015; 32: 199–206. .

